# Nectin-2 (CD112) Is Expressed on Outgrowth Endothelial Cells and Regulates Cell Proliferation and Angiogenic Function

**DOI:** 10.1371/journal.pone.0163301

**Published:** 2016-09-27

**Authors:** YeonSung Son, BomNaeRin Lee, Young-Jin Choi, Seon Ae Jeon, Ju-Hyun Kim, Hoo-Keun Lee, Sang-Mo Kwon, Je-Yoel Cho

**Affiliations:** 1 Department of Biochemistry, BK21 Plus and Research Institute for Veterinary Science, School of Veterinary Medicine, Seoul National University, Seoul, 151–742, Korea; 2 College of Pharmacy, Gachon University, Incheon 406–840, Republic of Korea; 3 Laboratory for Vascular Medicine & Stem Cell Biology, Medical Research Institute, Department of Physiology, school of Medicine, Pusan National University, Yangsan, 626–870 Korea; Centro Cardiologico Monzino, ITALY

## Abstract

Outgrowth endothelial cells (OECs) are a subpopulation of endothelial progenitor cells (EPCs) that have the capacity for proliferation and the ability to promote angiogenesis. In this study, we identified Nectin-2 as a surface protein of OECs through unbiased quantitative proteomics analysis. Using immunocytochemistry and flow cytometry, we confirmed that Nectin-2 is highly expressed on OECs. Nectin-2 (CD112) expression was limited or lower on mononuclear cells (MNCs) and mature tube-forming endothelial cells (ECs). Blocking Nectin-2 with a neutralizing monoclonal antibody significantly increased the trans-well migration and tube forming capacity of OECs. Similarly, Nectin-2 knockdown resulted in enhanced tube formation, cell migration and proliferation with p-Erk activation. Moreover, Nectin-2 deficiency resulted in compensatory increase of other Nectin family genes including Nectin-3 and Necl-4 which promote VEGFR signaling. These results indicate that Nectin-2 is a surface marker and an important regulator of OECs, with significant implications for the isolation of OECs and blocking Nectin-2 on OECs by an antibody for angiogenic applications.

## Introduction

Endothelial progenitor cells (EPCs) are a subpopulation of mononuclear cells found in peripheral or cord blood. EPCs were first identified in 1997 [1]: the researchers described the isolation of CD34+ cells from human peripheral blood by using magnetic microbeads. Since this initial report, a number of groups have isolated EPCs from peripheral blood, bone marrow, fetal liver and umbilical cord blood. Previous studies used cell surface molecules such as CD34, CD133, VEGFR-2 (KDR/FLK-1/CD309), VE-Cadherin (CD144), Tie-2, etc., or combination of multiple molecules [2–6].

After the first report [1], subsequent studies identified a subpopulation of EPCs that were named outgrowth endothelial cells (OECs) or circulating or late EPCs, and endothelial colony-forming cells (ECFCs). OECs display remarkable potential for proliferation and for promoting angiogenesis [7]. Unlike early EPCs, which are another subpopulation of EPCs that arise early in the culture of mononuclear cells and display heterogeneous non-proliferating characteristics, OECs arise 14 days after culture of mononuclear cells and display homogenous and distinctive proliferation characteristics. Moreover, OECs have shown greater migration potential and capillary tube-forming capacity than early EPCs. For this reason, OECs have led to a new paradigm for therapeutic neovascularization strategies in the regeneration of ischemic cardiac tissues and blood vessels [8].

However, despite many studies on the role of OEC in various human disorders, OEC-based cell therapies have struggled. These difficulties are due in part to the lack of good surface markers for OECs. Several previous studies have indicated that therapeutic success depends on better isolation and evaluation of the target cells. To date, only a few proteins are used to isolate and evaluate OECs. For example, endothelial markers, such as VEGFR-2, VE-Cadherin, CD31 (PECAM-1), and CD34, have also served as OEC markers. However, none of these molecules have proven useful as OEC markers. In this report, we identified Nectin-2 as a novel surface marker of OECs by mass spectrometry (MS)-based proteomics analysis.

Nectin-2, a poliovirus receptor-related 2 protein, is a type I transmembrane glycoprotein and a member of the Ig gene superfamily. Nectin-2, also known as CD112, is an adhesion molecule involved in the formation of cell junctions and interactions with other Nectin-family molecules. Nectin-2 is known to trans-interact with various Nectin-like (Necl) molecules such as Nectin-1, Nectin-3, PVR (Necl-5), and DNAM-1 (CD226) [9–11]. Nectin-2 also interacts with different scaffold proteins and is indirectly linked to the E-cadherin system. Nectins regulate multiple cellular functions such as cell movement, proliferation, polarization, survival, differentiation and cell adhesion by interacting with various proteins. For example, in the testis, Nectin-2 is likely to be essential for the formation of Sertoli-cell-spermatid junctions with Nectin-3 [3, 12, 13]. However, despite numerous reports concerning the role of Nectin-2, its expression and role in OECs have yet to be studied. In this report, we investigated the biological role of Nectin-2 in OECs and show that Nectin-2 is a novel surface marker of OECs, and regulates OEC migration, proliferation and angiogenesis.

## Materials and Methods

### Culture of human outgrowth endothelial cells (OECs) and human umbilical vein endothelial cells (HUVECs)

hUCB for OECs and HUVECs were collected from healthy volunteers after obtaining informed consent from all subjects according to the protocol approved by the Institutional Review Board of the Pusan National University Yangsan Hospital, Republic of Korea (Approval No. PNUH-2012-19). Briefly, total mononuclear cells were isolated by Ficoll (GE healthcare, Buckinghamshire, U.K) gradient density centrifugation of hUCB. Freshly isolated cells were cultured in endothelial growth medium-2 (EGM-2) supplemented with 5% fetal bovine serum, human vascular endothelial growth factor, human basic fibroblast growth factor, human epidermal growth factor, human insulin-like growth factor 1, ascorbic acid, and GA-1000 (Lonza, Walkersville, MD, USA). After 4 days in culture, non-adherent cells were discarded and attached cells were cultured further. Cultures were subjected to long-term culture to allow for the formation of spindle-shaped colonies and media was replenished for 14–21 days. OECs were then trypsinized and expanded over several passages at a splitting ratio of 1:2. OECs used for this study ranged from passage 5 to 11. HUVECs were used as previously described [14].

### LC-MS/MS-based glycoproteomics analysis

Total cell lysates or membrane fractions were collected from OECs and HUVECs. The cell pellet from two 145 cm^2^ dishes was washed twice with PBS, resuspended in a hypotonic buffer (20 mM KCl, 10 mM HEPES, pH 7.4) for 2 min, and collected again by centrifugation (1000 g, 5 min). The resulting pellet was re-suspended in sucrose homogenization buffer (255 mM sucrose, 20 mM HEPES, 1 mM EDTA, 10 mM Tris-HCl pH 7.4 plus protease inhibitors) by up and down strokes in a 25-G needle. The number of strokes was determined empirically during preliminary experiments under the described conditions by observing via phase-contrast microscopy when more than 95% of the cells were broken. The cell lysates were transferred to microfuge tubes and centrifuged at 14,000 g for 10 min. Then the resulting supernatant was transferred to 4 ml polycarbonate tubes and centrifuged at 245,000 g for 2 hr. The pellets were then re-suspended in 100 mM Na_2_CO_3_ (pH 11) and disrupted mechanically using a 25-G syringe, before incubating with rotation for a further 90 min at 245,000 g. Each preparation from two 145 cm^2^ dishes of cells yielded approximately 50 mg of protein.

Proteins were extracted from cell lysates or membrane fractions using a Covaris S-series (Covaris, USA) in lysis buffer containing 8 M Urea and 0.1 M Tri–HCl, pH 8.5. Protein concentration was measured using the BCA Protein Assay Kit (Thermo, USA). Two-hundred fifty micrograms of proteins from cell lysates or membrane fractions were used in this study, and 30 kDa Microcon filtration devices (Millipore, Ultracel YM-30, Billerica, MA, USA) were used for detergent removal and protein digestion following filter-aided sample preparation procedures (FASP). Peptides were eluted from the filter with 0.5 M NaCl. After the sample pH was adjusted to approximately pH 2–3, the sample was desalted using a C18 spin column (Harvard Apparatus, Holliston, MA, USA) and dried completely using a centrifugal concentrator (SCANVAC, LaboGene Aps, Lynge, Denmark). Approximately 250 μg of digested peptides were mixed with lectin solution containing a combination of concanavalin A (ConA) and wheat germ agglutinin (WGA) (Sigma Aldrich, USA), resulting in mixtures of peptides and lectins with a mass proportion of 1: 2. The mixtures were transferred to new YM-30 filter units (Millipore, Ultracel YM-30, Billerica, MA, USA). After a 1 hr incubation at room temperature, the unbound peptides were eluted by centrifugation. The captured peptides were washed, followed by deglycosylation with peptide-N-glycosidase F (Roche, Indianapolis, IN, USA). After incubation for 12 hr at 37°C, the deglycosylated peptides were eluted and purified on a C18 spin column (Harvard Apparatus, Holliston, MA, USA) and then dried by vacuum centrifugation.

Each samples were then resuspended in 20 μl of water with aqueous 0.1% formic acid (FA) for LC–MS/MS analysis and separated in a capillary column packed with C18 at a flow rate of 0.3 μl/min. Mobile phase A (0.1% FA in H_2_O) and mobile phase B (90% acetonitrile, 0.1% FA in H_2_O) were used to establish the 90-min gradient of 3–45% B (0–60 min), 45–90% B for 60–60.01 min, 90% B for 60.01–75 min, 90–10% B, 75–75.01 min, and 10–3% B for 75.01–90 min. The peptide samples were analyzed by 6550 Accurate-Mass Q-TOF LC/MS (Agilent Technologies, DE, USA) at an electrospray potential of 1.8 kV. A drying gas flow of 11 L/min and a fragmentor at 175 V were used. The Q-TOF was set to perform data acquisition in the positive mode, such that an m/z range of 100–3000 was used in the MS and MS/MS scan. Each sample was subjected to LC–MS three times for reproducibility.

MS/MS data from the Q-TOF instrument were converted to .mzXML format by Trapper 4.3.1 (TPP, ISB, USA) for database searching in SEQUEST (Sorcerer 3.5; Sage-N Research, La Jolla, CA, USA). Search parameters were as follows: precursor mass tolerance, 20 ppm; product ion mass tolerance, 50 ppm; 2 missed cleavages allowed; semi tryptic peptides; fixed modification of carbamidomethyl cysteine; and variable modifications of oxidized methionine, N-terminal carbamylation and a deamidated asparagine. Spectra with a PeptideProphet and ProteinProphet probability value > 0.9 (significance threshold error rate < 0.01) and a valid glycosylation consensus sequence N-X-S/T (X is not proline) were considered for further manual evaluation.

### Immunocytochemistry

For immunocytochemistry, OECs were cultured on 1% gelatin-coated cover slips in a 12 well plate, washed with Ca^2+^/Mg^2+^ PBS, and then fixed in 4% paraformaldehyde for 15 min at 4°C. After five washes, the plate was blocked for 1 hr with 10% normal serum in Ca^2+^/Mg^2+^ PBS at room temperature. The plate was incubated with anti-CD31, anti-CD144 (VE-Cadherin) (BD Pharmingen), or anti-Nectin-2 (1:100; BioLegend, USA) antibody for 1 hr at room temperature and washed five times. The bound primary antibody was detected with fluorescein-conjugated anti-mouse IgG for 1 hr at room temperature. After six washes with Ca^2+^/Mg^2+^ PBS, OECs were stained with 4,6 diamidino-2-phenylindole (DAPI, 0.25 μg/mL) in PBS containing 0.1% Triton X-100. Then, after another four washes with Ca^2+^/Mg^2+^ PBS, OECs were mounted with fluorescence mounting medium (Dako; Produktionsvej, Denmark). Fluorescence images acquired using an Olympus IX71 camera (Olympus Corporation; Tokyo, Japan).

### RT-PCR and real-time quantitative RT-PCR Analysis

Total RNA was isolated from OECs and HUVECs using the High Pure RNA Isolation kit (Roche Applied Science). RNA was treated with DNase I for each sample, followed by PCR amplification using the Access RT-PCR System Kit (Promega). First-strand cDNAs were reverse transcribed with avian myeloblastosis virus reverse transcriptase (Promega) at 42°C for 45 min. PCR reactions were performed in a thermocycler (Bio-Rad) with cycling parameters as follows: denaturation at 94°C for 30 sec, annealing at 57°C for 30 sec, and elongation at 72°C for 1 min. A final extension at 72°C for 10 min was terminated by rapid cooling at 4°C. Cycling times were determined for each primer set to be within the exponential phase of amplification. PCR products were run on 1.2% agarose gels containing 400 ng/ml ethidium bromide and visualized on a UV transilluminator. Sense and antisense primers were designed to amplify the cDNAs encoding CD14, CD45, CD105, CD117, VE-cadherin (CD144), CD146, VEGFR-2 (CD309), Nectin-1/2/3 and Necl-1/4. Quantitative RT-PCR was performed using the SYBR Green PCR Kit (Qiagen) and analyzed with CFX (Bio-Rad). The name and sequences of the semi-quantitative and quantitative RT-PCR primers for each pair are listed in Table 1.

**Table 1:** Quantitative real-time PCR primers.

Gene	Sequence (5’—3’)	Length
Nectin-1	CAACTACCACATGGACCGCT TGCCAGGCTGTAGTTGATGG	250
Nectin-2	CGGAACTGTCACTGTCACCA GACACTTCAGGAGGGTAGCG	150
Nectin-3	CACTGGAAAACCCGTTGCAC CGGATGTCCTTTTCCAAGGC	186
Necl-1	ACAAGGCTTAACCCGGGAAG AATCCGAGTGAGCTTTCCCC	218
Necl-4	GCCGTCTGCACCAGTATGAT GTGGTGGGTGTCTTCTGTGT	209
Tie 2	GGCCAAGTTCTTAAGGCGCGC GGGCGCGTACTCAATGGCCA	213
CD105	TCACCACAGCGGAAAAAGGT CAGGAACTCGGAGACGGATG	162
c-Kit (CD117)	TTATTCCTGACCCCAAGGCG AGACACAACAGGCACAGCTT	161
CD146 (MCAM)	TGGGCGCTATGAATGTCAGG CGAGGTCCTGGCTACTCTCT	176
VE-Cadherin (CD144)	ATCACAGATGTGGACGAGCC AGAACTGGCCCTTGTCACTG	169
VEGFR2 (KDR, CD309)	GGAGGACTTCCAGGGAGGAA TCTCCCGACTTTGTTGACCG	151
vWF	ATGGTCCGGCATGAGAACAG ACACACATGGTCTGTGCAGTT	150
CD14	TCCCTCAATCTGTCGTTCGC GGATTCCCGTCCAGTGTCAG	152
CD45	AAACGGAGATGCAGGGTCAA CCTCTTCCATTGACGGCCAG	218
GAPDH	ATCCCATCACCATCTTCCAG CCATCACGCCACAGTTTCC	170

### Analysis of cell cycle phases and multi-color immunofluorescence by flow cytometry

Cells were fixed overnight with 70% cold ethanol and washed with PBS containing 1% horse serum before being subjected to DNA staining. Fixed cells were pelleted and washed in ice-cold PBS, and cellular DNA was stained with 50 μg/mL propidium (Sigma) solution containing Triton-X-100 (Sigma), 0.1% sodium citrate (Sigma), and 100 μg/mL RNase A (Roche). The fluorescent cells were sorted after 1 h of incubation at RT in the dark. Cellular DNA content was analyzed by flow cytometry using a FACS Calibur (BD Biosciences, San Jose, CA, USA) equipped with an argon laser, and data were evaluated using CellQuest 3.0.1 software (Becton-Dickinson, Franklin Lakes, NJ). At least 25,000 cells were used for each analysis. Cell cycle progression and forward scatter are displayed as histograms and pie charts. The proportion of non-apoptotic cells in different phases of the cell cycle was then recorded at least in triplicate and expressed as the mean ± SD.

For multiple color flow cytometric analyses, cells were incubated with appropriate primary antibodies, phycoerythrin (PE)-conjugated antibody and fluorescein-conjugated antibody for 30 min at 4°C. After two washes with PBA (0.5% BSA in PBS), propidium iodide (PI)-negative cells were analyzed for antibody binding using the FACSCalibur (BD Immunocytometry System) and CellQuest software (BD Immunocytometry System).

### Tubulogenesis, migration (wound-healing) and Boyden-chamber assays

Tube formation assays on Matrigel were performed as described previously (Shota et al, 2015). Briefly, 96-well culture plates were coated with 70 μl of growth factor-reduced Matrigel per well. OECs were seeded on the coated plates at a density of 2.5×10^4^ cells per well in EGM-2 plus 2% FBS in the presence of 40 ng/ml VEGF, followed by incubation at 37°C for 24 hr.

Migration assays were also performed as described previously (Shota et al, 2015). Briefly, OECs and HUVECs growing on 12-well plates coated with 1% gelatin were scratched with a sterile 200 μl pipette tip. The cells were cultured for 12 hr in EGM-2 plus 2% FBS.

Boyden-chamber migration of OECs and HUVECs was performed as previously described (Shota et al, 2015). Briefly, OECs and HUVECs in 100 μl medium were added to the top chambers of 24-well transwell plates (5.0 μm pore size; Costar). EGM-2 plus 2% FBS in the presence 40 ng/ml of VEGF was added to the lower chambers. After 24 hr of incubation at 37°C with 5% CO_2_, images were acquired of the bottom wells.

### Western Blotting

Western blotting was performed as previously reported (Ref). Briefly, OECs and HUVECs were washed twice with ice-cold PBS and lysed in lysis buffer (RIPA buffer, Thermo) with protease inhibitors. Anti-CD112 (Nectin-2) antibodies were used at 1:2,000 and anti-mouse HRP conjugated secondary antibodies at 1: 4,000. The signals were detected using an ECL Plus kit (GE Healthcare, USA).

### Statistical analyses

The data were expressed as the mean values ± standard error. The data were analyzed by Excel software, and a *t*-test or ANOVA was used to compare the data pertaining to the different experimental groups. Differences were considered significant when the *P* values were <0.01.

## Results

### Characterization of outgrowth endothelial progenitor cells (OECs)

**Fig 1:**
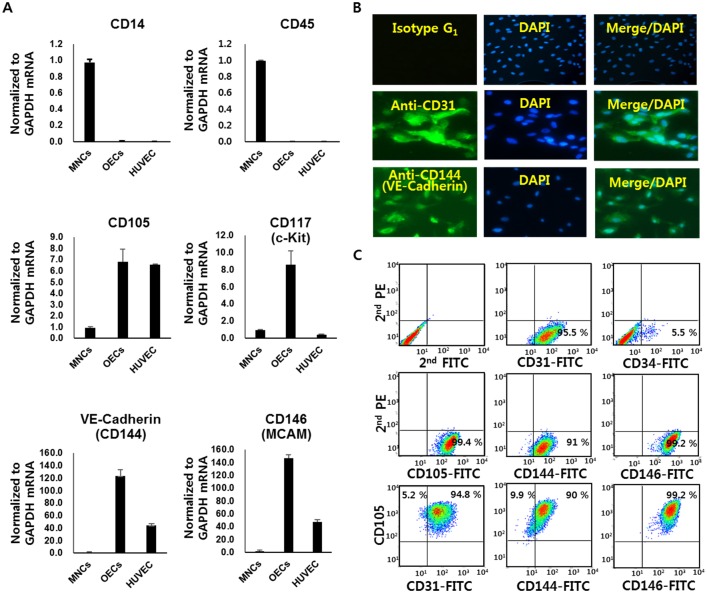
Characterization of outgrowth endothelial progenitor cells (OECs). **(A)** Quantitative real-time RT-PCR analysis of mRNA expression in MNCs, OECs and HUVECs. OECs and HUVECs expressed the endothelial cell markers, CD105, CD117 (c-Kit), CD144 (VE-cadherin)and CD146 (MCAM) but do not express the hematopoietic cell markers CD14 and CD45 (*P* < 0.01). **(B)** Immunofluorescence reveals that OECs were positive for anti-human CD31-FITC and anti-human CD144-FITC antibodies. Nuclei are stained blue with DAPI. **(C)** FACS analysis of OECs cell-surface-stained with the common endothelial markers CD105, CD144 (VE-cadherin) and CD146.

To characterize the isolated outgrowth endothelial cells (OECs), we performed real-time quantitative RT-PCR, immunocytochemistry and flow cytometry analyses of MNCs and OECs. The expression of endothelial and stem cell markers in OECs was analyzed in comparison with the expression profiles of MNCs and HUVECs. Our data demonstrated that the hematopoietic cell marker CD45 and the monocyte/macrophage marker CD14 were completely negative in the OECs. However, endothelial markers such as CD105, VE-Cadherin (CD144) and CD146 were highly expressed compared with MNCs. CD117 (c-Kit), a hematopoietic marker, VE-Cadherin and CD146 showed higher expression in OECs than in HUVECs (Fig 1A).

In addition to quantitative RT-PCR, immunocytochemistry studies were performed to characterize the OECs. The expression of endothelial markers CD31 (PECAM-1) and CD144 (VE-cadherin), but not isotype IgG1, was detected on the surface OECs (Fig 1B). Furthermore, double-labeling flow cytometry analyses were performed at the single-cell level. OECs were analyzed for CD105-PE/CD31-FITC, CD105-PE/CD144-FITC and CD105-PE/CD146-FITC. Double-labeling for CD105-PE/CD146-FITC revealed homogeneous populations, with over 99% of OEC cells being double positive. In the CD105-PE/CD31-FITC analysis, the main population was CD31-positive (94%), but approximately 5% of cells were CD105-positive. Additionally, CD105-PE/CD144-FITC analysis showed that the main population of OECs tested was CD144-positive, but 10% were CD105-positive (Fig 1C). Additionally, the cell-surface marker expression of the OECs derived from different cord-blood sources were analyzed by flow cytometry. The expression levels of endothelial surface markers differed depending on the donor (S1A–S1C Fig).

### Nectin-2 is highly expressed in OECs

**Fig 2:**
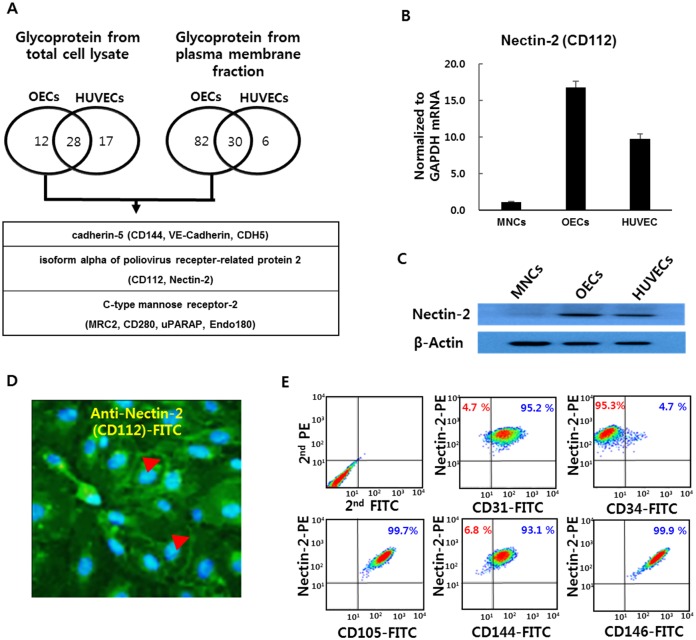
Nectin-2 is strongly expressed in OECs. **(A)** Mass-spectrometric identification of glycoproteins expressed on OECs and HUVECs. Venn diagram showing cell-surface proteins detected only in OECs via analyses of glycoproteins from both total cell lysates and membrane fractions. **(B)** Nectin-2 mRNA levels expressed as median percentages relative to a housekeeping marker (*P* < 0.01). **(C)** Nectin-2 protein expression in MNCs. OECs and HUVECs were analyzed by western blotting using an anti-human Nectin-2 antibody. **(D)** Immunostaining without permeabilization. OECs were stained with anti-Nectin-2-FITC conjugated mAb. Arrowheads show the signal for Nectin-2 on the surface of OECs. **(E)** FACS analysis of cell-surface Nectin-2 and endothelial cell markers. Representative FACS analysis of OECs double stained with each of the following antibodies: Nectin-2 (CD112), common endothelial cell markers (CD105, CD144 (VE-Cadherin), CD146, CD31) and an endothelial progenitor marker (CD34).

To identify cell-surface markers of OECs, we performed a proteomics-based survey to identify differentially expressed proteins on the surface of OECs and HUVECs. We prepared both total cell lysates and membrane fractions for this proteome analysis. Because glycoproteins are the most abundantly expressed cell-surface markers, we first enriched the glycoproteins of the total cell lysates and membrane fractions by lectin based glyco-capture. In the total cell lysate analysis, a total of 57 glycoproteins were identified (40 for OECs; 45 for HUVECs). In the plasma membrane fraction, a total of 118 glycoproteins were identified (112 for OECs; 36 for HUVECs) (Fig 2A). We tallied the proteins that were selectively expressed at high levels in OECs but not in HUVECs for each method and then pooled the common proteins from both methods. Three proteins remained as OEC-selective cell-surface membrane glycoproteins: Cadherin-5 (CD144, VE-Cadherin), Nectin-2 (CD112) and MRC-2 (CD280). CD144 (VE-Cadherin) is known to be expressed on both OECs and HUVECs, but was identified in our analysis because it was expressed at a marginally detectible level in OECs (spectral counts 2 or 4) but not in the HUVECs. In this study, we focused on Nectin-2, whose function has not been reported previously in the OEC.

We next compared the expression of Nectin-2 in mononuclear cells (MNCs), OECs and HUVECs. Among the 3 cell types, Nectin-2 showed the highest expression in OECs at both the transcript (Fig 2B) and protein levels (Fig 2C). Nectin-2 transcript level was very low and the protein level was almost no detectible in MNCs (Fig 2B and 2C, S2 Fig). However, unlike the mass spectrometry data, both transcript and protein levels of Nectin-2 were considerably high in HUVECs, although the levels in HUVECs were lower than in OECs (S3 Fig). This again might be due to the mass spectrometry-detectable marginal level differences of Nectin-2 protein expression between OECs and HUVECs. Immunocytochemistry using an anti-Nectin-2-FITC antibody demonstrated that Nectin-2 was densely displayed on the cell surface of OECs (Fig 2D). In addition, we tested a panel of surface markers by flow cytometry. OECs were co-stained by Nectin-2 together with the EC surface markers (Fig 2E). As shown in Fig 2E, 95.2%, 99.7%, 93.1% and 99.9% of the Nectin-2-positive OECs were positive for the expression of CD31, CD105, CD144 (VE-Cadherin) and CD146, respectively. These data again showed that, indeed, the Nectin-2 was co-expressed with the EC markers such as CD31 and CD144, and most strikingly with CD146 and CD105, previously known OEC/HUVEC markers. However, only 4.7% of OECs co-expressed Nectin-2 and CD34, an EPC marker. Thus, it seems that Nectin-2 is likely to be highly expressed on OECs and HUVECs, but not on CD34-positive EPCs. It is known that HUVECs are similar to OECs in an aspect that they have also greater proliferative potential than do adult endothelial cells (ECs). Unlike vWF and Tie2, which show increasing expression upon serial passaging of OECs, our data showed Nectin-2 levels decreased after serial passaging of OECs (S3 Fig). These results suggest that high levels of Nectin-2 can serve as a potential cell surface marker for OECs and HUVECs.

### Nectin-2 inhibits OEC migration and tube formation

**Fig 3:**
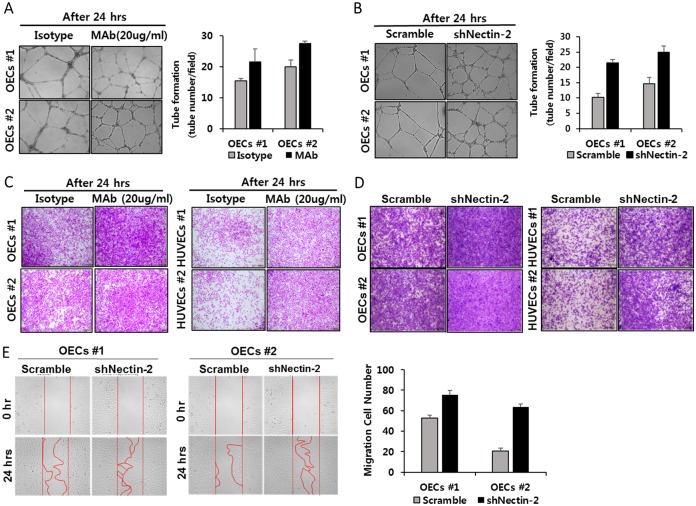
Enhanced migration and tube formation induced by inhibitory anti-Nectin-2 monoclonal antibody and Nectin-2 knockdown. **(A)** Increased tube formation by anti-Nectin-2 mAb treatment. Anti-Nectin-2 mAb-treated OECs and control OECs were cultured in Matrigel with 20 ng/ml VEGF, and the number of tubes was counted (*P* < 0.01). **(B)** Increased tube formation by Nectin-2 knockdown. OECs were infected with scrambled shRNA or Nectin-2 shRNA. After a 12-h incubation with shRNA, Puromycin selection was performed with 0.5 μg/ml Puromycin and continued for 3 days (p < 0.01). **(C)** Boyden-chamber assays were used to assess the migratory potential of OECs treated with anti Nectin-2 mAb. Nectin-2 mAb treatment increased OEC migration. **(D)** Scramble shRNA- or Nectin-2 shRNA-treated OECs were assessed by Boyden-chamber assays. Nectin-2 shRNA increased OEC and HUVEC migration compared with the scramble shRNA control. **(E)** Representative images of in vitro OEC scratch-wound healing assays. Nectin-2 knockdown or control OECs were seeded on gelatin-coated (1% gelatin) plates. Upon reaching confluency, cell layers were scratched with a pipette tip. Photos were taken immediately after wounding at 0 hr and 24 hr and the number of tubes was counted (*P* < 0.01).

To examine the function of Nectin-2, a blocking monoclonal antibody against Nectin-2 was used to disrupt its function in OECs. OECs were treated with anti-Nectin-2 mAbs (20 μg/ml), and their tube forming capacity was subsequently measured in a Matrigel tube-formation assay. OECs have the capacity to form tube structures in EGM-2 media upon VEGF treatment. OECs treated with the blocking Nectin-2 mAb and Nectin-2 knockdown shRNA showed enhanced capillary formation, as measured by tube number and the time of tube appearance after VEGF treatment (Fig 3A and 3B). Several adhesion molecules have been reported to regulate tube formation. DICAM knockdown also exhibited the formation of a more extensive capillary network, but Necl-5 knockdown showed decreased network formation [15, 16]. Next, we measured the effect of Nectin-2 inhibition on cell migration. The anti-Nectin-2 mAb treatment significantly promoted OEC migration, as measured in Boyden-chamber assays (Fig 3C). A similar pattern was also observed in HUVECs treated with this mAb. These results suggest that Nectin-2 might have a negative effect on the tube formation and migration of OECs. Next, to examine the role of Nectin-2 in cell migration, Nectin-2 was knocked down in OECs. Boyden-chamber assays revealed that Nectin-2 knockdown significantly increased the migration of OECs and HUVECs (Fig 3D). Similarly, wound-healing cell migration assays also showed increased migratory ability in Nectin-2 knockdown OECs (Fig 3E). These results are in accordance with the anti Nectin-2 mAb treatment results and suggest that Nectin-2 inhibits OECs cell migration.

### Nectin-2 inhibits OECs proliferation

**Fig 4:**
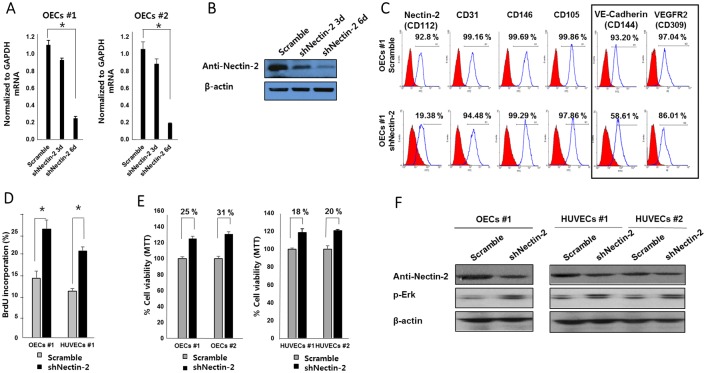
Nectin-2 knockdown increases OEC proliferation. OECs were infected with scramble shRNA or Nectin-2 shRNA lentivirus. **(A)** Nectin-2 mRNA expression levels were analyzed by quantitative RT-PCR (*P* < 0.01). **(B)** Nectin-2 protein expression was analyzed by western blotting. **(C)** Representative FACS analysis of Nectin-2-knockdown in OECs. Cell-surface expression of VE-Cadherin (CD144) and VEGFR-2 was reduced by Nectin-2 knockdown. Other endothelial cell-surface markers were not changed in OECs. Cell proliferation was evaluated by BrdU and MTT incorporation. (D) The percentage of BrdU incorporation (mean±SD) was analyzed for scramble and Nectin-2 shRNA in both OECs and HUVECs (*P* < 0.01). (E) MTT assays performed on OECs and HUVECs (*P* < 0.01). (F) Nectin-2 knockdown increased phospho-ERK activity. Nectin-2 was knocked down in OECs and HUVECs with shRNA, and the knockdown efficiency was monitored by anti-CD112 (Nectin-2) MAb in Western Blot.

We next examined the effect of Nectin-2 down-regulation by generating a Nectin-2-knockdown OEC line. Specific depletion of Nectin-2 was accomplished using lentivirus-mediated transduction and expression of sequence-specific short hairpin RNA (shRNA). Infection with Nectin-2 shRNA lentivirus significantly reduced Nectin-2 mRNA expression levels to 22±2% and 18±5% in the two OEC lines (Fig 4A). Nectin-2 protein levels were also markedly downregulated in Nectin-2 shRNA-transfected OECs compared to that in control shRNA-transfected cells (Fig 4B). Targeted Nectin-2-knockdown cells were also applied to flow cytometry analysis. When Nectin-2 expression was knocked-down with three independent shRNAs, the Nectin-2 population has clearly reduced from 92.8% to 19.4%. Flow cytometry analysis showed that Nectin-2 knockdown did not alter CD31, CD105 or CD146 endothelial cell marker expression. However, the VE-Cadherin and VEGFR-2-expressing cell populations were reduced from 93.20% to 58.61% and from 97.04% to 86.01%, respectively (Fig 4C). Similar results were obtained in HUVECs (S4A and S4B Fig). Endothelial cells express cell-type-specific transmembrane adhesion proteins such as VE-Cadherin at Adherens junctions (AJs) and Claudins and Occludin and JAMs at tight junctions (TJs) [17, 18]. The Nectin-Afadin system has been reported to play an important role in both AJ and TJ organization. In the Nectin-Afadin complex, Ponsin binds to Afadin, Vinculin and alpha-Catenin and thereby helps to anchor the complex to Actin. VE-Cadherin forms a complex with VEGFR-2 [17]. Therefore, our results indicate that Nectin-2 may regulate the expression levels of VE-Cadherin and VEGFR-2 in the Actin complex.

Next, we investigated the effect of Nectin-2 on OEC proliferation. The mitotic indexes, as measured by bromodeoxyuridine (BrdU) incorporation, were significantly increased in Nectin-2 knockdown cells compared with control cells (Fig 4D). Cell viability, as determined by MTT, was also significantly increased in Nectin-2 knockdown OEC cells (Fig 4E). Similar results were obtained in HUVECs (S4C Fig). These results indicate that Nectin-2 also inhibits the proliferation of OECs. Then, we examined the activation of ERK1/2 signaling, which is important for cell proliferation and migration. Indeed, Nectin-2 knockdown increased phospho-ERK1/2 levels in HUVECs as well as in OECs (Fig 4F).

### Association of Nectin-2 and other Nectin family members

**Fig 5:**
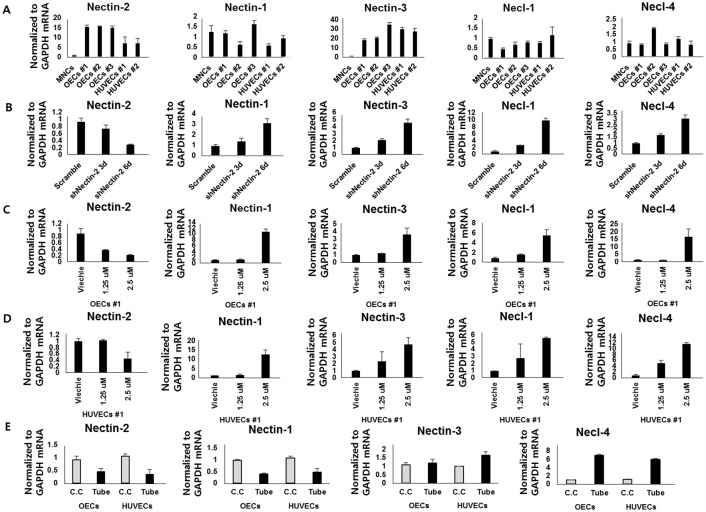
Compensation of Nectin-2 function by other Nectin family members and related genes. **(A)** Expression of the Nectin family genes Nectin-1, 2 and 3 and the related genes Necl-1 and Necl-4 was measured by real-time RT-PCR in MNCs, OECs and HUVECs. **(B)** Nectin-family and related gene expression levels were tested in Nectin-2-knockdown OECs. **(C)** Nectin-2 mRNA levels were assessed 24 hrs after CdCl_2_ treatment in OECs and **(D)** in HUVECs. **(E)** OECs were cultured in Matrigel for 24 h, and the resulting tube-forming OECs were harvested and evaluated for Nectin and Nectin-like molecule expression (*P*< 0.01).

We then investigated whether Nectin-2 has effects on Nectin family members and related genes. First, the expression of Nectin family members and Necl-1 (CDAM1) and 4 (CADM4) in OECs was compared with the expression profiles in MNCs and HUVECs by real-time PCR (Fig 5A). Previous reports have shown that Nectin-3 and Necl-4 (CADM4) are expressed in endothelial cells, whereas Nectin-1 not [19, 20]. Our results showed that Nectin-3, as well as Nectin-2, was markedly expressed in OECs and HUVECs compared with MNCs. However, Nectin-2 knockdown increased the expression of all Nectin family members and Necls (Nectin-1, Nectin-3, Necl-1 and Necl-4) compared with the sh-control in OECs (Fig 5B). On the other hand, Nectin-2 knockdown in HUVECs showed an opposite pattern for Nectin-1 and Necl-4, a similar change for Necl-1 and no change in Nectin-3 (S5 Fig). To further test the effect of Nectin-2 on Nectin-2 family genes, OECs were treated with CdCl_2_, which is known to reduce Nectin-2 gene transcription by halting the binding of transcription factors on its promoter region (Fig 5B). CdCl_2_ exposure resulted in a dose-dependent reduction in Nectin-2 mRNA levels in OECs (S6A Fig) and HUVECs (S6B Fig). Nectin-2 protein levels were also reduced by 1.25-μM CdCl_2_ treatment for 24 hr (S6C Fig). Again, downregulation of Nectin-2 by CdCl_2_ resulted in a significant increase in Nectin-family genes, Nectin-1, Nectin-3, Necl-1, and Necl-4, in OECs and HUVECs (Fig 5C and 5D), confirming the shRNA-mediated knockdown experiments. This expression increase might partly serve to compensation for Nectin-2 function following knockdown.

Then, we tested whether the Nectin family are affected by *in vitro* tube formation. As shown in Fig 5E, Nectin-1 and Nectin-2 mRNA levels were markedly reduced in tube formation condition of OECs and HUVECs. Interestingly, however, Necl-4 levels were markedly increased in tube formation of OECs and HUVECs and Nectin-3 levels were slightly increased in tube formation condition of HUVECs. These data also suggest that Necl-4 and Nectin-3 might have opposite functions to Nectin-2 upon tube forming condition.

## Discussion

Outgrowth endothelial cells (OECs), also known as late endothelial progenitor cells (LEPCs), are renowned for promoting vascular repair and angiogenesis [5, 21–23]. I The present study, we employed proteomics combined with glycoprotein enrichment to both OECs and HUVECS to identify OEC cell surface markers and identified Nectin-2 as a surface marker that is highly expressed on OECs. We then confirmed Nectin-2 expression via quantitative real-time PCR, immunocytochemistry and flow cytometry. However, consecutive analysis showed that Nectin-2 co-localizes with common endothelial cell surface markers CD31, CD105, VE-Cadherin and CD146 (Fig 2E). Therefore, our results suggest that Nectin-2 is a candidate cell-surface marker for endothelial cell-featured OECs. Moreover, we found that Nectin-2 expression is very limited in MNCs, which are early-stage cells that can differentiate into different cell lineages. On the other hand, we also showed the Nectin-2 (CD112) is expressed not only in OECs but also in HUVECs at lower levels. From our characterization of Nectin-2 in comparison with other surface markers between OECs and HUVECs, we observed that it is hard to differentiate OECs from HUVECs because they share most of the cell surface markers including Nectin-2. It is of note that HUVECs are similar to OECs in an aspect that they have also greater proliferative potential than do adult endothelial cells. However, Nectin-2 expression is limited in tube-forming ECs that are in the final stage of EC differentiation and are unable to proliferate. Our findings showed Nectin-2 can serve as a novel surface marker candidate for the isolation and enrichment of circulating OECs for research and therapeutic purposes.

Nectin-2 belongs to the Nectin family, which are Ca^2+^-independent Ig-like cell adhesion molecules. Nectin-family molecules contain three Ig-like domains in their extracellular regions [10]. These three Ig-like domains are reported to form homo- and hetero-dimers with other Nectins, playing a pivotal role in cell-cell adhesion and migration [24]. Similar to other Nectin family members, our study shows that Nectin-2 is involved in the regulation of cell-cell interactions and motility. However, unlike other Nectin molecules such as Nectin-3 and Necl-5 [3, 25], Nectin-2 inhibits the migration of OECs, as inhibiting Nectin-2 with an anti-Nectin-2 mAb enhanced OEC migration. Knocking down Nectin-2 with shRNA similarly increased the migration of OECs. Nectin-2 also inhibits tube formation by OECs. This conclusion is supported by the observation that Nectin-2 levels are low in ECs during tube formation and that antibody blocking and Nectin-2 knockdown promoted tube formation. Taken together, these results suggest that Nectin-2 on OECs inhibits cell migration and terminal differentiation into tube-forming endothelial cells. Therefore, it is expected that blocking Nectin-2 on OECs with a therapeutic antibody might promote the cell migration and angiogenesis. This observation is also supported by reports that DICAM (Dual Ig domain-containing adhesion molecule), another cell-to-cell adhesion molecule, may participate in endothelial cell adhesion and migration and consequently in the regulation of angiogenesis. DICAM knockdown resulted in a more extensive capillary network. During angiogenesis, DICAM is known to be involved in the inhibition of angiogenesis [3, 20, 26]. On the other hand, our data also showed that Necl-4 was up-regulated upon Nectin-2 knockdown. Necl-4 was reported to localize to the leading edges of migrating cells. Interestingly, both cell proliferation and tubulogenesis were significantly reduced in Necl-4-knockdown ECs [20], suggesting that Necl-4, by contrast, promotes both EC proliferation and tube formation.

The physiologic functions of Nectins involve several downstream signaling events that are related to vascular endothelial growth factor receptor (VEGFR-2) in human umbilical endothelial cells [27–29]. VEGFR-2 is known to be regulated by direct interactions with several cell adhesion proteins such as Nectin-3, VE-Cadherin and Integrin in angiogenesis and cell proliferation [30]. VE-Cadherin is a specific adhesion molecule that plays a vital role in the maintenance and control of endothelial cell-cell interactions. In addition, VE-Cadherin can limit cell proliferation by retaining VEGFR-2 at the membrane, preventing its internalization into signaling compartments [17, 18, 25]. Meanwhile, Nectin-2 is also likely to be physically connected to VE-Cadherin and VEGFR-2. The notion that Nectin-2 physically interacts with VE-Cadherin or VEFGR-2 is also supported by our results that Nectin-2 knockdown reduced VE-Cadherin and VEGFR-2 levels in OECs and HUVEC (Fig 4C, S4B Fig). However, it needs further studies to see whether the decrease of VEGFR-2 and VE-cadherin affects the cell physiology of OECs upon Nectin-2 knock-down.

The increased proliferation OECs following Nectin-2 knockdown was also associated with increased p-Erk, which is a strong cell proliferation signal (Fig 4H). The activated ERK (p-ERK) in Nectin-2 knockdown cells might be caused by compensation effects. We found that Nectin-2 deficiency resulted in compensatory increase of other Nectin family genes. In Nectin-2 knockdown cells, the upregulation of Nectin-1, Nectin-3 and Necl-4 was observed. Similar results were also obtained in CdCl_2_ (a Nectin-2 blocker)-treated OECs (Fig 5B and 5C). This compensation effect might partially explain the negative effects of Nectin-2 on proliferation, migration and tube formation in OECs because Nectins, especially Nectin-3 and Necl-4, are known to promote VEGFR-mediated signaling. Interestingly, Nectin-3 and Necl-4 expression was also higher in tube-forming endothelial cells (Fig 5E). However, these results do not exclude the possibility that an unknown signaling mechanism underlies regulation of OEC proliferation, migration and angiogenesis by Nectin-2.

OECs have been shown to circulate in the blood and participate in angiogenesis either by producing cytokines in a paracrine way or by differentiating into mature endothelial cells [31]. Considering the renowned proliferative and angiogenic character of OECs, it seems contradictory that Nectin-2 is highly expressed in OECs because we found that Nectin-2 negatively regulates OECs’ function. However, our data demonstrate that Nectin-2 expression is decreased in OECs that are participating in tube formation. This result suggests that Nectin-2 functions in quiescent OECs circulating in the peripheral blood but not in OECs that are actively participating in angiogenesis. Further studies are required to reveal exactly how Nectin-2 is involved in the suppression of OEC proliferation and differentiation into endothelial cells.

## Supporting Information

S1 Fig Characterization of various OECs by FACS analysis. Flow cytometric analysis of various OECs labeled with antibodies against common endothelial markers (CD34, CD31, VE-Cadherin (CD144) and VEGFR-2 (CD309). Endothelial cells expressed all commonly accepted EC markers, but the markers were differentially expressed on the cells from different donors. PE indicates phycoerythrin; FITC, fluorescein isothiocyanate
(TIF)

S2 Fig Flow cytometry analysis of the expression of CD14 (hematopoietic surface marker) and Nectin-2 (CD112) by MNCs derived from donor cord blood. Nectin-2 was weakly expressed on MNCs.
(TIF)

S3 Fig Nectin-2 expression pattern following serial passage culture of OECs. Nectin-2 showed high expression levels in OECs compared with MNCs and HUVECs. EC marker expression on OECs during continuous passage was monitored by quantitative PCR. vWF and Tie-2 increased, whereas Nectin-2 expression decreased in serial passage culture (*P*< 0.01).
(TIF)

S4 Fig Downregulation of Nectin-2 activity by Nectin-2 knockdown in HUVECs. HUVECs were infected with scramble shRNA or Nectin-2 shRNA. After 12-h incubation with shRNA, puromycin selection at 0.5 μg/ml was initiated and continued for 3 days. Following puromycin selection, the stable cells were analyzed further. **(A)** Nectin-2 mRNA expression levels analyzed by quantitative RT-PCR (*P*< 0.01) and **(B)** representative FACS analysis of Nectin-2-knockdown in HUVECs. VE-Cadherin (CD144) and VEGFR-2 showed reduced cell surface expression following Nectin-2 knockdown. The expression of other endothelial cell surface markers was unchanged.
(TIF)

S5 Fig Nectin-related gene expression after Nectin-2 knockdown by shRNA in HUVECs. When Nectin-2 expression was down-regulated HUVECs, Nectin-1 and Necl-4 were down-regulated, Necl-1 was up-regulated, and Nectin-3 levels did not change (*P*< 0.01).
(TIF)

S6 Fig Dose-dependent Nectin-2 downregulation in CdCl_2_-treated OECs and HUVECs. The mRNA levels of Nectins and Nectin-like molecules were determined after CdCl_2_ treatment in (A) OECs and (B) HUVECs to investigate the compensation effect of Nectin-2 down-regulation (*P*< 0.01). (C) Proteins were extracted from CdCl_2_-treated OECs and HUVECs, and western blotting was performed using an anti-Nectin-2 antibody.
(TIF)
