# Supplementary figures and images for: Nectin-2 (CD112) Is Expressed on Outgrowth Endothelial Cells and Regulates Cell Proliferation and Angiogenic Function
Source: PLoS One. 2016 Sep 27;11(9):e0163301. doi: 10.1371/journal.pone.0163301 (PMC5038973; doi:10.1371/journal.pone.0163301)

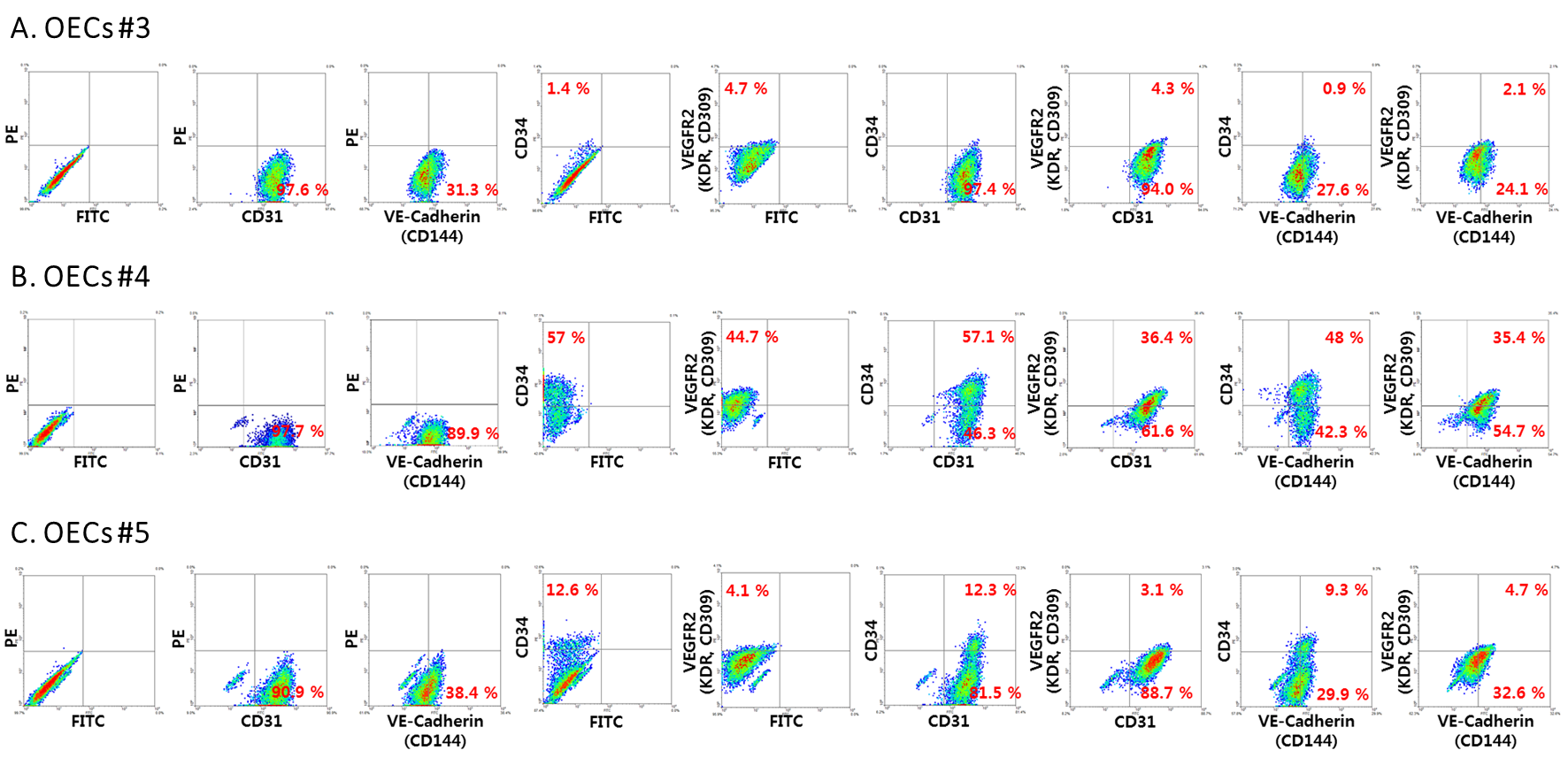

Supplement: S1 Fig — Flow cytometric analysis of various OECs labeled with antibodies against common endothelial markers (CD34, CD31, VE-Cadherin (CD144) and VEGFR-2 (CD309). Endothelial cells expressed all commonly accepted EC markers, but the markers were differentially expressed on the cells from different donors. PE indicates phycoerythrin; FITC, fluorescein isothiocyanate (TIF) [file pone.0163301.s001.tif]

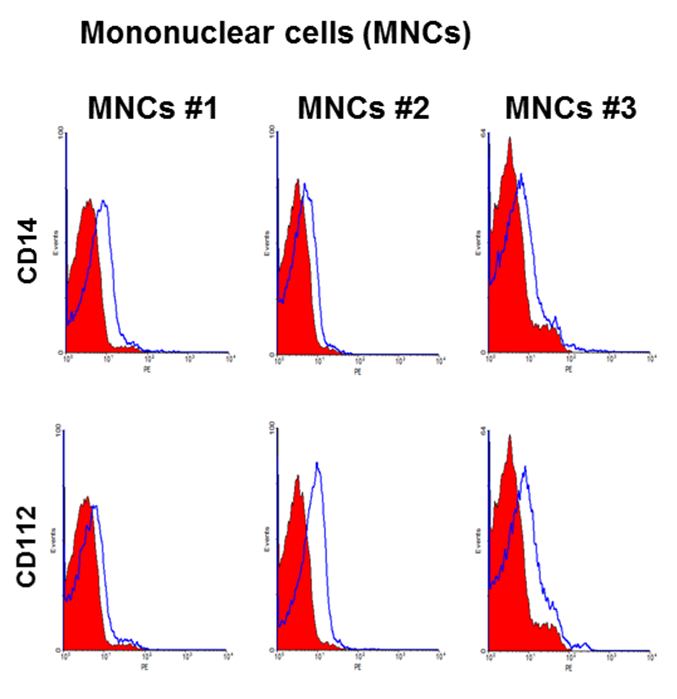

Supplement: S2 Fig — Nectin-2 was weakly expressed on MNCs. (TIF) [file pone.0163301.s002.tif]

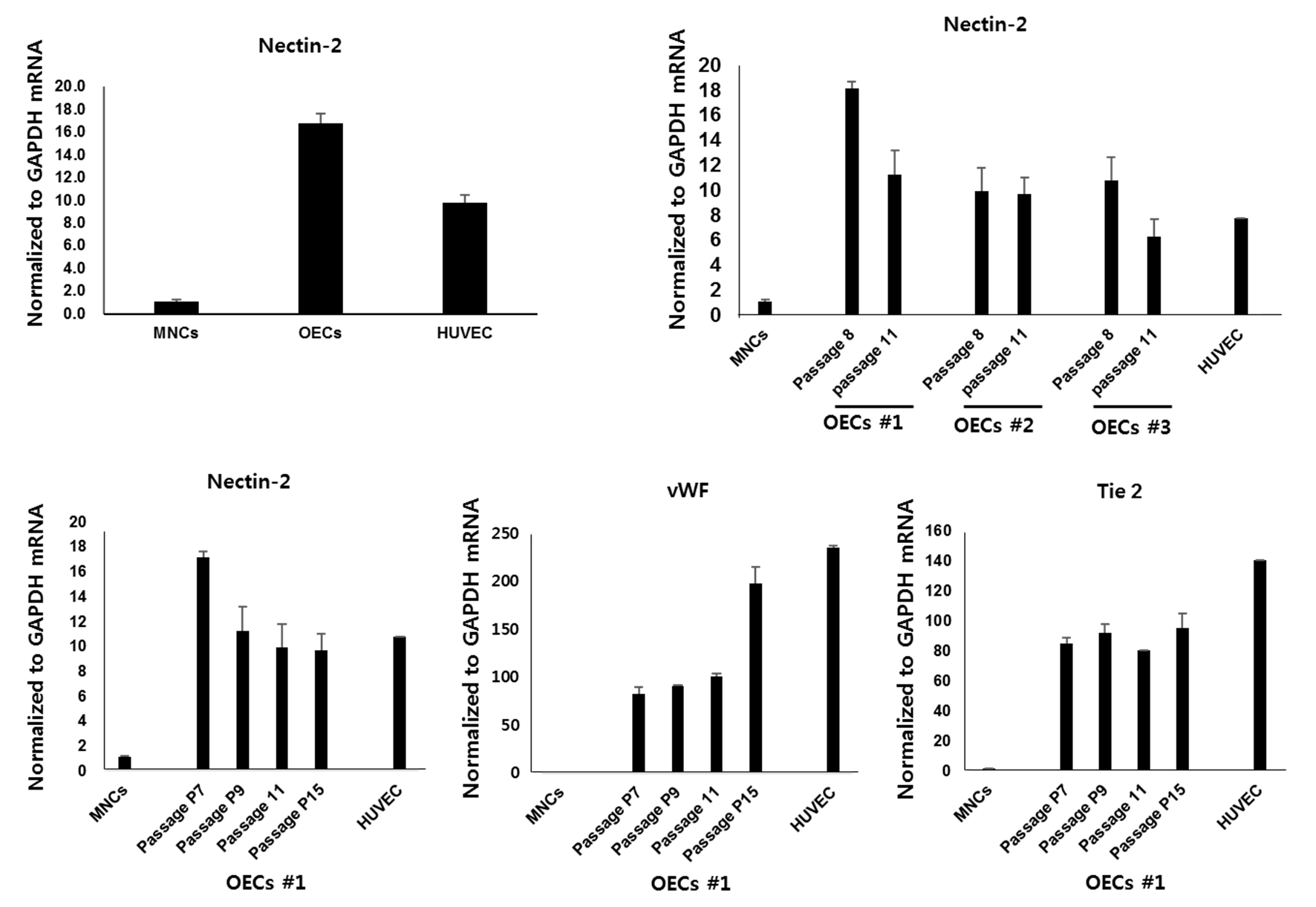

Supplement: S3 Fig — Nectin-2 showed high expression levels in OECs compared with MNCs and HUVECs. EC marker expression on OECs during continuous passage was monitored by quantitative PCR. vWF and Tie-2 increased, whereas Nectin-2 expression decreased in serial passage culture (P< 0.01). (TIF) [file pone.0163301.s003.tif]

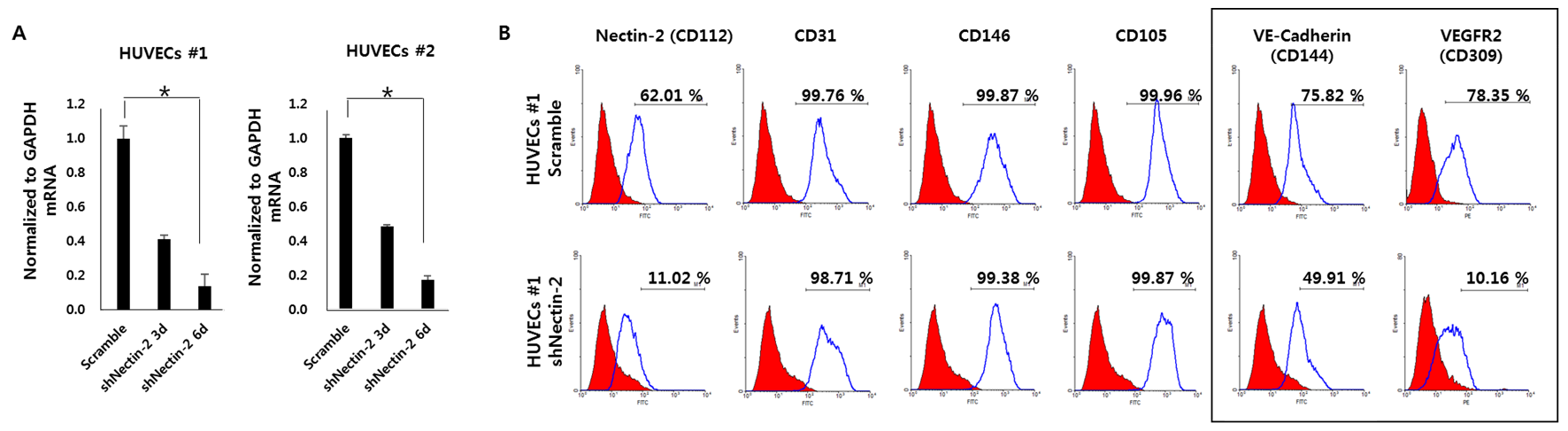

Supplement: S4 Fig — HUVECs were infected with scramble shRNA or Nectin-2 shRNA. After 12-h incubation with shRNA, puromycin selection at 0.5 μg/ml was initiated and continued for 3 days. Following puromycin selection, the stable cells were analyzed further. (A) Nectin-2 mRNA expression levels analyzed by quantitative RT-PCR (P< 0.01) and (B) representative FACS analysis of Nectin-2-knockdown in HUVECs. VE-Cadherin (CD144) and VEGFR-2 showed reduced cell surface expression following Nectin-2 knockdown. The expression of other endothelial cell surface markers was unchanged. (TIF) [file pone.0163301.s004.tif]

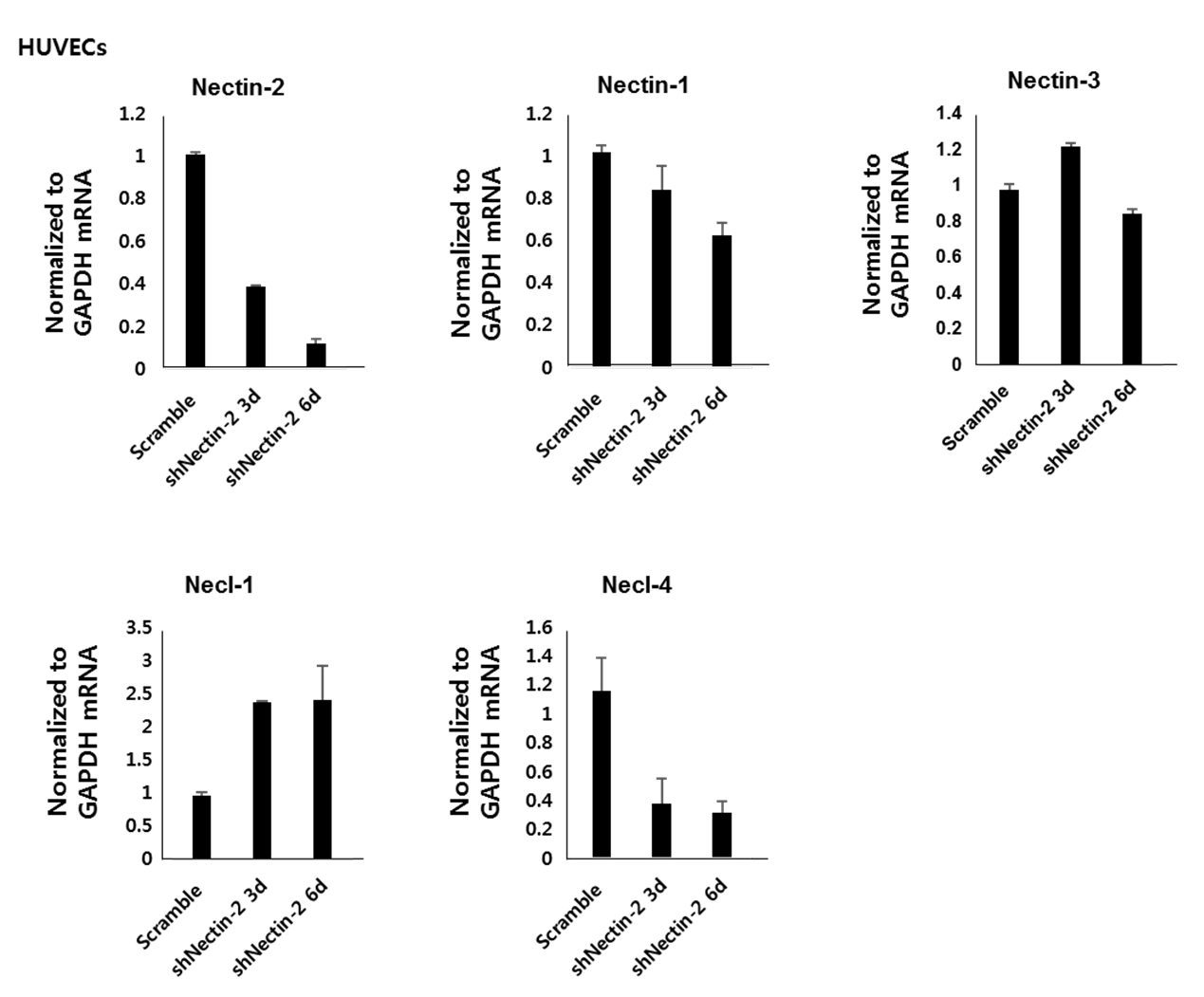

Supplement: S5 Fig — When Nectin-2 expression was down-regulated HUVECs, Nectin-1 and Necl-4 were down-regulated, Necl-1 was up-regulated, and Nectin-3 levels did not change (P< 0.01). (TIF) [file pone.0163301.s005.tif]

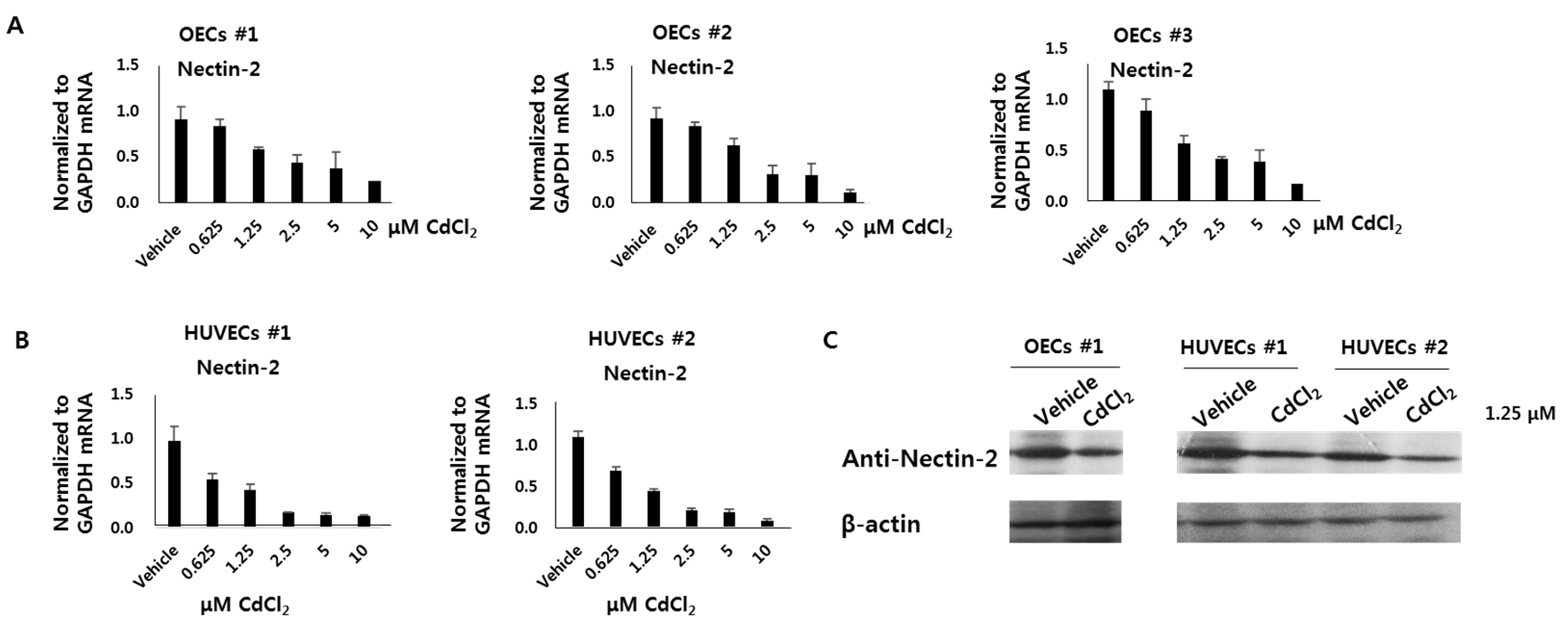

Supplement: S6 Fig — The mRNA levels of Nectins and Nectin-like molecules were determined after CdCl2 treatment in (A) OECs and (B) HUVECs to investigate the compensation effect of Nectin-2 down-regulation (P< 0.01). (C) Proteins were extracted from CdCl2-treated OECs and HUVECs, and western blotting was performed using an anti-Nectin-2 antibody. (TIF) [file pone.0163301.s006.tif]
